# Outcomes After Receipt of Neuraxial or Regional Anesthesia Instead of General Anesthesia for Lower Limb Revascularization Surgery: Protocol for a Systematic Review and Meta-analysis

**DOI:** 10.2196/32170

**Published:** 2021-11-26

**Authors:** Derek J Roberts, Hannah Dreksler, Sudhir K Nagpal, Allen Li, Jeanna Parsons Leigh, Timothy Brandys, Prasad Jetty, Luc Dubois, Henry T Stelfox, Daniel I McIsaac

**Affiliations:** 1 Division of Vascular and Endovascular Surgery Department of Surgery University of Ottawa Ottawa, ON Canada; 2 Clinical Epidemiology Program Ottawa Hospital Research Institute Ottawa Hospital Ottawa, ON Canada; 3 School of Health Administration Faculty of Health Dalhousie University Halifax, NS Canada; 4 Division of Vascular Surgery Department of Surgery Western University London, ON Canada; 5 Department of Epidemiology and Biostatistics Faculty of Medicine Western University London, ON Canada; 6 Department of Critical Care Medicine University of Calgary Calgary, AB Canada; 7 O’Brien Institute for Public Health University of Calgary Calgary, AB Canada; 8 Department of Anesthesiology and Pain Medicine University of Ottawa Ottawa, ON Canada

**Keywords:** epidural anesthesia, lower limb revascularization surgery, neuraxial anesthesia, peripheral artery disease, spinal anesthesia, vascular surgery, anesthesia, surgery, limb, nerves, spine, outcome, protocol, review, artery, cardiovascular, hospital

## Abstract

**Background:**

Patients undergoing lower limb revascularization surgery for peripheral artery disease (PAD) have a high risk of perioperative morbidity and mortality and often have long hospital stays. Use of neuraxial or regional anesthesia instead of general anesthesia may represent one approach to improving outcomes and reducing resource use among these patients.

**Objective:**

The aim is to conduct a systematic review and meta-analysis to determine whether receipt of neuraxial or regional anesthesia instead of general anesthesia in adults undergoing lower limb revascularization surgery for PAD results in improved health outcomes and costs and a shorter length of hospitalization.

**Methods:**

We will search electronic bibliographic databases (MEDLINE, EMBASE, the seven databases in Evidence-Based Medicine Reviews, medRxiv, bioRxiv, and Google Scholar), review papers identified during the search, and included article bibliographies. We will include randomized and nonrandomized studies comparing the use of neuraxial or regional anesthesia instead of general anesthesia in adults undergoing lower limb revascularization surgery for PAD. Two investigators will independently evaluate the risk of bias. The primary outcome will be short-term (in-hospital or 30-day) mortality. Secondary outcomes will include longer-term mortality; major adverse cardiovascular, pulmonary, renal, and limb events; delirium; deep vein thrombosis or pulmonary embolism; neuraxial or regional anesthesia–related complications; graft-related outcomes; length of operation and hospital stay; costs; and patient-reported or functional outcomes. We will calculate summary odds ratios (ORs) and standardized mean differences (SMDs) using random-effects models. Heterogeneity will be explored using stratified meta-analyses and meta-regression. We will assess for publication bias using the Begg and Egger tests and use the trim-and-fill method to estimate the potential influence of this bias on summary estimates. Finally, we will use Grading of Recommendations, Assessment, Development, and Evaluation (GRADE) methodology to make an overall rating of the quality of evidence in our effect estimates.

**Results:**

The protocol was registered in the International Prospective Register of Systematic Reviews (PROSPERO). We executed the peer-reviewed search strategy on March 2, 2021. We completed the review of titles and abstracts on July 30, 2021, and plan to complete the review of full-text papers by September 30, 2021. We will complete full-text study data extraction and the risk-of-bias assessment by November 15, 2021, and conduct qualitative and then quantitative data synthesis and GRADE assessment of results by January 1, 2022, before drafting the manuscript. We anticipate that we will be able to submit the manuscript for peer review by the end of February 2022.

**Conclusions:**

This study will synthesize existing evidence regarding whether receipt of neuraxial or regional anesthesia instead of general anesthesia in adults undergoing lower limb revascularization surgery for PAD results in improved health outcomes, graft patency, and costs and a shorter length of hospital stay. Study results will be used to inform practice and future research, including creation of a pilot and then multicenter randomized controlled trial.

**Trial Registration:**

Prospero CRD42021237060; https://www.crd.york.ac.uk/prospero/display_record.php?RecordID=237060

**International Registered Report Identifier (IRRID):**

PRR1-10.2196/32170

## Introduction

### Background

Lower limb revascularization surgeries (ie, endarterectomy, patch angioplasty, and arterial bypass) are commonly performed across North America [1-4]. In the United States, at least 15,000 to 20,000 lower limb arterial bypass surgeries are performed annually, while an average of 1650 lower limb revascularization surgeries are performed per annum in Ontario (Canada’s most populous province) [1,5,6]. Although endovascular therapy is increasingly used for treatment of chronic limb-threatening ischemia (CLTI), defined as peripheral artery disease (PAD) manifested by rest pain or tissue loss, and some patients with lifestyle-limiting vasculogenic intermittent claudication, it is less durable than surgical revascularization and not suitable for some patients’ anatomical pattern of disease [1,7]. There is also equipoise among many clinicians as to whether endovascular or surgical revascularization should be offered to patients with PAD who are candidates for both [1,7].

Patients undergoing lower limb revascularization surgery for PAD are typically older (average age of approximately 70 years), are current or past cigarette smokers, and have several comorbidities that place them at high risk for perioperative morbidity and mortality [2,3]. These include diabetes and coronary artery disease, chronic obstructive pulmonary disease, and chronic kidney disease [2,3]. Compared to adults with coronary artery or cerebrovascular disease, those with PAD have a higher risk of cardiovascular events, hospitalization, and hospitalization for coronary, carotid, or lower limb revascularization [8]. Those patients undergoing lower limb revascularization surgery for PAD also often require long postoperative (and sometimes preoperative) hospital stays and consume substantial health care system resources [9,10]. Hospitalization costs for those with PAD exceed those for patients with coronary artery or cerebrovascular disease, with lower limb revascularization procedures accounting for a substantial amount of these costs [8].

Use of neuraxial (spinal or epidural) or regional (peripheral nerve block) anesthesia instead of general anesthesia may represent one approach to improving postoperative health outcomes and reducing resource use among patients undergoing lower limb revascularization surgery for PAD [1,2,11,12]. Both neuraxial and regional anesthesia improve peripheral circulation and avoid mechanical ventilation, while neuraxial anesthesia improves coagulation and blunts surgical stress responses [1,13-17]. A 2013 Cochrane systematic review of four small randomized controlled trials (RCTs) reported that patients who received neuraxial instead of general anesthesia for lower limb revascularization surgery had a lower pooled risk of pneumonia [18]. An increasing number of large nonrandomized comparative studies have also recently reported that use of neuraxial or regional anesthesia (instead of general anesthesia) is associated with variable reductions in adjusted perioperative cardiopulmonary and renal complications, lengths of hospital stay, in-hospital or short-term mortality, and health care system costs [1,3,11,12,19]. However, reported findings of these studies are inconsistent and some remain limited by a risk of residual confounding by indication [1]. This type of confounding occurs because anesthetic techniques may be chosen based on patient, provider, and hospital characteristics, and these characteristics predict subsequent health and health care system outcomes [1,20].

### Objectives

We propose to conduct a systematic review and meta-analysis of randomized and nonrandomized comparative studies to determine whether receipt of neuraxial or regional anesthesia instead of general anesthesia in adults undergoing lower limb revascularization surgery for PAD results in improved health outcomes, graft patency, and costs and a shorter length of hospital stay. We will also determine whether results of these studies vary by differences in study design, included patient populations, or risk of bias.

## Methods

### Protocol and Role of the Sponsor

This systematic review protocol was developed and reported according to the Preferred Reporting Items in Systematic Reviews and Meta-Analyses (PRISMA) 2020 statement [21] and the Meta-analysis Of Observational Studies in Epidemiology (MOOSE) proposal [22]. It is reported according to the PRIMSA protocols (PRISMA-P) statement (see Multimedia Appendix 1 for the completed PRISMA-P checklist) [23,24]. The protocol is registered in the International Prospective Register of Systematic Reviews (PROSPERO; registration no. CRD42021237060). The University of Ottawa had no role in the development of the protocol.

### Focused Clinical Question

We formulated the focused clinical question for the study according to the patient, intervention (or exposure, for nonrandomized studies), comparison, outcome, and design (PI[E]COD) method of designing clinical questions for systematic reviews. Our focused clinical question was:

- P: for adults (≥18 years of age) undergoing lower limb revascularization surgery for PAD
- I(E): does receipt of neuraxial (spinal or epidural) or regional (peripheral nerve block) anesthesia (as the primary anesthetic technique)
- C: compared with general anesthesia (as the primary anesthetic technique), including general anesthesia combined with other anesthesia techniques
- O: Result in improved outcomes, including (1) primary outcome (short-term [in-hospital or 30-day] mortality) and (2) secondary outcomes (longer-term mortality; major adverse cardiovascular, pulmonary, renal, and limb events; delirium; deep vein thrombosis or pulmonary embolism; neuraxial or regional anesthesia–related complications; graft-related outcomes; length of operation and hospital stay; costs; and patient-reported or functional outcomes [see below for definitions of these outcomes])
- D: in randomized and nonrandomized comparative studies?

### Information Sources

We will search MEDLINE; MEDLINE Epub Ahead of Print, In-Process & Other Non-Indexed Citations; EMBASE; the seven databases contained within Evidence-Based Medicine Reviews (American College of Physicians [ACP] Journal Club®; the Cochrane Central Register of Controlled Trials, the Database of Systematic Reviews, and the Methodology Register Database; the Database of Abstracts of Reviews of Effects; the Health Technology Assessment Database; and the National Health Service Economic Evaluation Database); and medRxiv, bioRxiv, and Google Scholar from their first available date until study initiation without language, publication date, or other restrictions. To identify additional citations, we will also use the PubMed “related articles” feature and search bibliographies of included studies and relevant review papers identified during the search.

### Search Strategy

A vascular surgeon and epidemiologist with graduate training in information science and evidence synthesis methods (author DJR) created the initial MEDLINE search strategy. Using a combination of Medical Subject Heading (MeSH) terms and keywords, search filters were constructed covering the themes *anesthetic type* and *lower limb revascularization surgery*. With assistance from a medical librarian, this strategy was then piloted and refined by adding additional MeSH terms when new and relevant citations were located in iterative pilot searches. We then adapted the search for EMBASE by searching for Emtree terms covering subjects similar to the MeSH terms (see Tables 1 and 2 for our final electronic bibliographic database search strategies).

**Table 1:** Ovid MEDLINE database search strategies.

Search theme	MeSH^a^ search terms	Search term text words
Lower limb revascularization surgery	arterial occlusive disease/surgery OR blood vessel prosthesis OR blood vessel prosthesis implantation OR endarterectomy OR ischemia/surgery OR lower extremity/surgery OR peripheral arterial disease/surgery OR peripheral vascular diseases/surgery OR vascular surgical procedures	((iliofemoral OR femoral OR femoral artery*) adj3 (endarterectom* OR patch* OR repair*)) OR ((femoral-distal OR femoral distal OR femoral-popliteal OR femoral popliteal OR femoral-tibial OR femoral tibial OR infrageniculate OR suprageniculate OR infrainguinal OR lower extremity OR lower limb OR peripheral vascular) adj3 (arterial surg* OR arterial bypass* OR bypass* OR bypass graft* OR bypass surg* OR graft* OR intervention* OR revascularization* OR revascularization procedure* OR vascular bypass* OR vascular bypass surg* OR vascular graft* OR vein graft* OR prosthetic graft*))
Anesthetic type	Anesthesia OR anesthesia, endotracheal OR anesthesia, epidural* OR anesthesia, spinal* OR anesthesia, local* OR anesthesia, general* OR nerve block	((general OR regional OR neuraxial OR epidural OR spinal) adj3 (anesthe*)) OR epidural* OR nerve block* OR peripheral nerve block* OR spinal*

^a^MeSH: Medical Subject Heading.

**Table 2:** Ovid EMBASE database search strategies.

Search theme	Emtree search terms	Search term text words
Lower limb revascularization surgery	artery bypass OR blood vessel graft OR bypass surgery OR critical limb ischemia/surgery OR endarterectomy OR limb ischemia/surgery OR peripheral artery occlusive disease/surgery OR prosthetic vascular graft OR vascular surgery OR vein bypass	((iliofemoral OR femoral OR femoral artery*) adj3 (endarterectom* OR patch* OR repair*)) OR ((femoral-distal OR femoral distal OR femoral-popliteal OR femoral popliteal OR femoral-tibial OR femoral tibial OR infrageniculate OR suprageniculate OR infrainguinal OR lower extremity OR lower limb OR peripheral vascular) adj3 (arterial surg* OR arterial bypass* OR bypass* OR bypass graft* OR bypass surg* OR graft* OR intervention* OR revascularization* OR revascularization procedure* OR vascular bypass* OR vascular bypass surg* OR vascular graft* OR vein graft* OR prosthetic graft*))
Anesthetic type	Anesthesia OR general anesthesia OR epidural anesthesia OR nerve block OR regional anesthesia OR spinal anesthesia	((general OR regional OR neuraxial OR epidural OR spinal) adj3 (anesthe*)) OR epidural* OR nerve block* OR peripheral nerve block* OR spinal*

### Data Management and Selection Process

The titles and abstracts of citations identified during the search will be exported into EndNote X9 reference management software (Clarivate, Thomson Reuters Corporation, Fairfax, VA, USA). This software will then be used to remove identical duplicates from the citation list. Two investigators will subsequently independently review the titles and abstracts of all identified citations and select any paper deemed potentially relevant by either investigator for full-text review using Covidence (Covidence, Melbourne, Australia). Finally, these two investigators will review the full text of all potentially relevant citations and select studies for inclusion in the systematic review. Disagreements regarding study inclusion will be resolved via consensus or arbitration by a third investigator (DJR).

### Eligibility Criteria

#### Population

We will include studies where participants were adults (≥18 years of age) undergoing lower limb revascularization surgery for PAD. Lower limb revascularization surgery will be considered to include iliofemoral or femoral endarterectomy or patch angioplasty and iliofemoral or infrainguinal bypass (eg, femoral-popliteal or femoral-tibial bypass) [1]. We will exclude studies that (1) included patients who underwent lower limb revascularization surgery that utilized a suprainguinal source of inflow aside from the external iliac arteries (eg, aortofemoral or axillofemoral bypass) because these procedures require general anesthesia [25] or (2) included >20% of patients reported to undergo surgery for indications other than PAD (eg, aneurysms or trauma).

#### Intervention/Exposure and Comparison

The intervention (for randomized studies) or exposure (for nonrandomized comparative studies) of interest will include neuraxial or regional anesthesia as the primary anesthesia technique. Neuraxial anesthesia will be defined as spinal, epidural, or combined spinal-epidural anesthesia without general anesthesia, while regional anesthesia will be defined as use of a peripheral nerve block without general anesthesia. The comparison of interest will be general anesthesia (including general anesthesia in combination with neuraxial or regional anesthesia).

#### Outcomes

The primary outcome will be short-term (in-hospital or 30-day) mortality. Secondary outcomes will include (1) longer-term mortality (mortality beyond 30 days); (2) major adverse cardiovascular events (cardiovascular death, stroke, or myocardial infarction) [26]; (3) delirium; (4) postoperative pulmonary complications (pneumonia, unplanned or prolonged mechanical ventilation, or acute respiratory distress syndrome); (5) deep vein thrombosis or pulmonary embolism; (6) acute kidney injury or initiation of new dialysis; (7) major adverse limb events (acute limb ischemia or amputation) [27]; (8) arterial bypass graft-related outcomes (primary, primary assisted, and secondary patency) [28]; (9) neuraxial or regional anesthesia–related adverse events (epidural hematoma, spinal cord injury, intracranial hemorrhage, or peripheral nerve injury); (10) costs; (11) length of operation and hospital stay; and (12) patient-reported or functional outcomes. Primary, primary-assisted, and secondary arterial bypass graft patency will be defined according to the reporting standards of the Society for Vascular Surgery [28]. According to these standards, primary patency refers to patency obtained without a need for additional or secondary surgical or endovascular procedures (or the interval of time from the original intervention until any intervention performed to maintain or re-establish patency) [28]. Primary-assisted patency represents patency achieved with the use of additional or secondary surgical or endovascular procedures, as long as occlusion of the primary treated site has not occurred [28]. Finally, secondary patency is patency obtained with the use of an additional or secondary surgical or endovascular procedure after occlusion occurs [28].

#### Design

The included studies must be randomized or nonrandomized (ie, cohort, case–control, or comparative effectiveness) comparative (ie, with a comparator group) studies of the above interventions [29,30]. We will include abstracts of studies not published in full, if they reported sufficient detail to determine eligibility. We will exclude nonrandomized studies that did not control for confounding in their effect estimates using matching, regression, propensity scores, instrumental variables, or another method [31]. For the primary analysis, we will limit inclusion of nonrandomized comparative studies to those controlling for a minimum set of confounders, including age, sex, type of lower limb revascularization surgery, surgical urgency, cardiovascular and pulmonary comorbidities, diabetes, and cognitive status/dementia [2,19], as has been recommended by guidance documents on meta-analyses of nonrandomized studies [31].

#### Setting and Language

There will be no restrictions regarding the setting or language of the study.

### Data Items and Collection Process

The same two investigators will independently extract data using an electronic data extraction spreadsheet and tables piloted on a representative sample of three randomized and three nonrandomized studies. We will extract the following data from included studies (where applicable or reported): (1) year of publication, design, data source, and study country or setting; (2) patient recruitment period; (3) inclusion and exclusion criteria; (4) patient and procedural characteristics, including the types of lower limb revascularization surgeries performed and the urgency of and indication(s) for these procedures; (5) characteristics of the anesthetics provided to the intervention and comparison groups (ie, percentage of spinal and epidural anesthesia, types of medications administered into the spinal or epidural space, and types of peripheral nerve blocks and medications used for these blocks); (6) follow-up duration; and (7) study outcomes and their definitions (as reported by study authors). For reported study outcomes, we will extract event rates or odds ratios (ORs), with 95% confidence intervals (CIs); group means (with SDs), for continuous differences; and other relative or absolute effect measures describing one or more outcomes of interest between the groups (or we will calculate them from the data provided). For nonrandomized comparative studies, we will extract the most thoroughly adjusted effect estimates (and which confounding factors were adjusted for) when variably adjusted outcomes were reported [32]. Where necessary, authors of studies will be contacted for additional clarifying or outcome information. In randomized studies, we will extract outcomes analyzed according to an intent-to-treat principle.

### Risk-of-Bias Assessment

Two investigators will independently judge the risk of bias among the included RCTs using the Cochrane Collaboration tool [29,33]. This tool includes questions regarding random sequence generation, allocation concealment, blinding of participants and personnel, blinding of outcome assessment, incomplete outcome data, selective reporting, and other biases [29]. Using this tool, we will judge each RCT to be at low, unclear, or high risk of bias in each of the above domains.

The same two investigators will also judge the risk of bias among the included nonrandomized studies using the Risk of Bias in Non-randomised Studies – of Interventions (ROBINS-I) tool [30]. This tool includes questions regarding bias due to confounding, in selection of participants into the study, in classification of interventions, due to deviations from intended interventions or missing data, in measurement of outcomes, and in selection of the reported result [30]. We will answer each question using the response options yes, probably yes, probably no, no, and no information [30]. We will then use these judgments within each domain to make an overall domain-level judgment about the risk of bias, as is recommended by the ROBINS-I authors [30].

Discrepancies between investigators in study-level risk-of-bias assessments will be resolved by discussion and consensus between the investigators or arbitration by a third investigator (DJR).

### Data Synthesis

#### Qualitative Data Synthesis

Characteristics of the included studies (including year of publication, country of origin, patient recruitment periods, inclusion and exclusion criteria, interventions and comparisons, and follow-up durations) and their included patient populations (mean/median ages and types of lower limb revascularization surgeries performed) will be tabulated by study design (randomized or nonrandomized comparative) and types of comparisons (ie, neuraxial or regional anesthesia compared with general anesthesia). This will allow us to compare recruitment periods and determine whether potentially overlapping data may have been reported before performing randomized and nonrandomized comparative study meta-analyses. We will also tabulate results of risk-of-bias assessments by study design (randomized or nonrandomized).

#### Quantitative Data Synthesis and Statistical Analyses

We will use the OR (for dichotomous outcomes) and the standardized mean difference (SMD) (for continuous outcomes) as the summary measures of association when combining results of randomized and nonrandomized studies, respectively. When risk ratios (RRs) or hazard ratios (HRs) were reported instead of ORs by study authors, we will pool these measures of association separately by estimate type, as has been suggested [31].

Results of randomized and nonrandomized studies will be pooled separately by comparison type (ie, by whether neuraxial or regional anesthesia was compared to general anesthesia) in primary analyses using the method of restricted maximum likelihood (REML) [34]. This method will be chosen to estimate between-study variance in meta-analyses, as has been recommended over other methods based on results of simulation studies [34]. If overlapping or duplicate data were used in nonrandomized studies, we will include the study with the largest sample size that reported an adjusted measure of association in meta-analyses. To assess for interstudy heterogeneity in our pooled estimates, we will inspect forest plots, calculate Cochran *Q* homogeneity and *I*^2^ inconsistency statistics, and conduct tests of homogeneity (*P* value<.10 considered significant, given the low power of these tests) [35-37]. As suggested by Higgins et al, we will consider *I*^2^ statistics of >25%, >50%, and >75% to represent low, moderate, and high degrees of heterogeneity, respectively [36].

In the presence of low or greater interstudy heterogeneity, we will conduct prespecified subgroup analyses using random-effects models and meta-regression, with the summary OR for in-hospital mortality as the dependent variable. We will use the following predictor variables in an attempt to explain heterogeneity in these stratified analyses or meta-regressions: (1) study design (randomized vs nonrandomized comparative), (2) publication status (abstract vs full-text publication), (3) whether there was a low versus higher risk of bias related to random sequence generation or allocation concealment in randomized studies, (4) whether nonrandomized studies reported an OR or other measure of association that was adjusted using the minimum confounder set (to determine whether better-adjusted estimates more closely agree with those obtained from randomized studies) [31], and (5) the proportion of patients included in the study who had a combined general and neuraxial or regional anesthetic in combination with a general anesthetic or who underwent an emergent operation, underwent a groin-only lower limb revascularization surgery, or were diagnosed with coronary artery disease, diabetes, chronic kidney disease, or critical limb ischemia.

We will evaluate for the presence of small study effects potentially due to publication bias for each outcome by visually inspecting produced funnel plots and using Begg and Egger tests (*P* value<.05 considered significant) [38]. When evidence of small study effects exists, we will use the Duval and Tweedie “trim and fill” method to estimate the potential influence of this type of bias on our pooled estimates [39-41]. In this method, small outlying studies are first “trimmed” (removed until the funnel plot is symmetrical) and then the remaining symmetrical studies are used to re-estimate the “true” center of the plot [39-41]. The plot is then “filled” (the missing, outlying study results and their theoretical balancing counterparts are replaced around the new center), and a small study effect–adjusted center is recalculated.

Statistical analyses will be performed using Stata MP version 13.1 (Stata Corporation, College Station, TX, USA) by a trained meta-analyst [39-41].

### Confidence in Cumulative Evidence

We will use the Grading of Recommendations, Assessment, Development, and Evaluation (GRADE) methodology to make an overall rating of the quality of evidence in our effect estimates for the primary and secondary outcomes (ie, confidence that our effect estimates are correct) [42,43]. To do this, we will first assess the risk of bias, consistency, directness, precision, and risk of publication bias associated with the evidence for the primary and each secondary outcome [44-48]. The overall confidence in these effect estimates will then be adjudicated as high (“further research is very unlikely to change our confidence in the estimate of effects”), moderate (“further research is likely to have an important impact on our confidence in the estimate of effects and may change the estimate”), low (“further research is very likely to have an important impact on our confidence in the estimate of effects and is likely to change the estimate”), or very low (“uncertain about the estimate of effects”) [24].

### Data Sharing

We will provide the raw data included in meta-analyses at the point of publication.

## Results

We executed the peer-reviewed search strategy on March 2, 2021. We completed the review of titles and abstracts on July 30, 2021, and plan to complete the review of full-text papers by September 30, 2021. We will complete full-text study data extraction and the risk-of-bias assessment by November 15, 2021. Subsequently, we will conduct qualitative and then quantitative data synthesis and the GRADE assessment of the results by January 1, 2022, before drafting the manuscript. We anticipate that we will be able to submit the manuscript for peer review by the end of February 2022.

## Discussion

### Summary

Patients undergoing lower limb revascularization surgery for PAD are at high risk of serious postoperative adverse events and consume substantial health care resources. We hypothesize that avoidance of general anesthesia may represent an efficacious approach to improving their health outcomes and reducing resource use [1,2,11,12]. Use of neuraxial or regional anesthesia instead of general anesthesia could improve outcomes after lower limb revascularization surgery in several biologically plausible ways. Clinical and translational research suggest that neuraxial and regional anesthesia improve peripheral circulation and avoid mechanical ventilation, while neuraxial anesthesia improves coagulation and blunts surgical stress responses [1,13-17]. Avoidance of general anesthesia may also help to reduce pulmonary complications by avoiding airway manipulation and invasive or positive-pressure ventilation [18].

By evaluating a variety of relevant primary and secondary outcomes, our systematic review will help to identify which outcomes (if any) are likely to be improved after receipt of neuraxial or regional anesthesia instead of general anesthesia in patients undergoing lower limb revascularization surgery. After synthesizing the available evidence, we will use the GRADE methodology to assess the risk of bias, consistency, directness, precision, and publication bias associated with the evidence for each outcome. We will then use these assessments to rate the overall confidence in the cumulative evidence for each of these outcomes to inform current lower limb revascularization surgery practice. We will also identify important knowledge gaps not addressed by the current literature on this topic. These may include a lack of patient-reported, functional, and longer-term outcomes after use of different types of anesthesia in patients undergoing lower limb revascularization surgery.

To the best of our knowledge, no multicenter RCTs comparing neuraxial or regional anesthesia and general anesthesia in adults undergoing lower limb revascularization surgery have been reported. If none are identified and future RCTs comparing neuraxial or regional anesthesia and general anesthesia are required, the findings of our systematic review will inform their design. For example, patients undergoing these surgeries often have multiple and sometimes life-threatening comorbidities and take a number of different medications, including antiplatelets and anticoagulants, some of which contraindicate the use of neuraxial anesthesia [1]. Our systematic review will identify common and relevant inclusion and exclusion criteria from existing RCTs, while helping to determine which lower limb revascularization surgeries may be appropriate to serve as inclusion criteria for an RCT. As different lower limb revascularization surgeries have different anticipated operative durations (eg, femoral endarterectomy vs femoral-tibial bypass), different approaches to using neuraxial anesthesia may be required for different surgeries (eg, a spinal anesthetic may be used for shorter duration surgeries while a combined spinal-epidural anesthetic may be needed for longer surgeries).

### Conclusion

In summary, we propose to synthesize existing evidence regarding whether receipt of neuraxial or regional anesthesia instead of general anesthesia in adults undergoing lower limb revascularization surgery results in improved health outcomes, graft patency, and costs and a shorter length of hospital stay. Study results will be used to inform practice and future research, including creation of a pilot and then multicenter RCTs comparing neuraxial and general anesthesia in this patient population.

## Appendix

Multimedia Appendix 1 PRISMA-P (Preferred Reporting Items in Systematic Reviews and Meta-Analyses Protocols) checklist.

## Abbreviations

CLTI: chronic limb-threatening ischemia
GRADE: Grading of Recommendations, Assessment, Development, and Evaluation
HR: hazard ratio
MeSH: Medical Subject Heading
OR: odds ratio
PAD: peripheral artery disease
PI(E)COD: patient, intervention (or exposure), comparison, outcome, and design
PRISMA: Preferred Reporting Items in Systematic Reviews and Meta-Analyses
PRISMA-P: Preferred Reporting Items in Systematic Reviews and Meta-Analyses Protocols
RCT: randomized controlled trial
REML: restricted maximum likelihood
ROBINS-I: Risk of Bias in Non-randomised Studies – of Interventions
RR: risk ratio
